# DNA methylation modulates *H19* and *IGF2* expression in porcine female eye

**DOI:** 10.1590/1678-4685-GMB-2016-0194

**Published:** 2017-03-06

**Authors:** Dongxu Wang, Guodong Wang, Hao Yang, Haibo Liu, Cuie Li, Xiaolan Li, Chao Lin, Yuning Song, Zhanjun Li, Dianfeng Liu

**Affiliations:** 1 College of Animal Science, Jilin University, Changchun, China; 2 Department of Emergency, First Hospital, Jilin University, Changchun, Jilin, China; 3 State Key Laboratory of Biotherapy, Sichuan University, Chengdu, Sichuan, China; 4 Guangzhou Institutes of Biomedicine and Health, Chinese Academy of Sciences, Guangdong, Guangzhou, China

**Keywords:** H19/IGF2, gene expression, DNA methylation, parthenogenetic, pig

## Abstract

The sexually dimorphic expression of *H19*/*IGF2* is evolutionarily conserved. To investigate whether the expression of *H19*/*IGF2* in the female porcine eye is sex-dependent, gene expression and methylation status were evaluated using quantitative real-time PCR (qPCR) and bisulfite sequencing PCR (BSP). We hypothesized that *H19/IGF2* might exhibit a different DNA methylation status in the female eye. In order to evaluate our hypothesis, parthenogenetic (PA) cells were used for analysis by qPCR and BSP. Our results showed that *H19* and *IGF2* were over-expressed in the female eye compared with the male eye (3-fold and 2-fold, respectively). We observed a normal monoallelic methylation pattern for *H19* differentially methylated regions (DMRs). Compared with *H19* DMRs, *IGF2* DMRs showed a different methylation pattern in the eye. Taken together, these results suggest that elevated expression of *H19/IGF2* is caused by a specific chromatin structure that is regulated by the DNA methylation status of *IGF2* DMRs in the female eye.

## Introduction

The imprinted gene *IGF2* has been reported to be paternally expressed and it is a growth factor in mammals (Barlow *et al*., 1991). The *H19* gene, which is maternally expressed, encodes a non-coding RNA (Bartolomei *et al*., 1991; Brunkow and Tilghman, 1991; Gabory *et al*., 2006). Numerous studies have demonstrated that the parent-specific expression of *H19/IGF2* depends on differentially methylated regions (DMRs) (Park *et al*., 2011; Thorvaldsen *et al*., 2006). Studies have also shown that DMRs play an important role in the regulation of gene expression in the *H19/IGF2* cluster within promoters and enhancers (Braunschweig *et al*., 2011).

It has been reported that the *H19* DMRs in mice contain three CTCF-binding motifs (Han *et al*., 2008). Through CTCF binding to the DMRs, *IGF2* is inactivated and maternal *H19* is expressed. However, when that binding is prevented, paternal *IGF2* is expressed. This is known as parent-specific chromatin loops (Wei *et al*., 2005), and, as part of this mechanism, *IGF2* DMRs are considered an epigenetic switch that regulates *H19* and *IGF2* expressions.

*H19/IGF2* are reciprocally imprinted in most tissues, although they are expressed at different levels, a fact which contributes to sex-bias in the female mouse eye, but not in other tissues (Hudson *et al*., 2010; Park *et al*., 2009; Reinius and Kanduri, 2013). In this study, we investigated the expression of *H19* and *IGF2* in the eye of female pigs to detect sex-specific imprinting effects. We hypothesized that the DNA methylation patterns of the *H19* DMRs and *IGF2* DMRs interact in the eye, and that this might regulate *H19* and *IGF2* expression through a specific chromatin structure. To test this hypothesis, we analyzed the expression pattern and methylation status of *H19*/*IGF2* in parthenogenetic (PA) cells by qPCR and bisulfite sequencing PCR (BSP).

## Materials and Methods

### Ethics statement

Pig experiments were carried out in accordance with the guidelines on animal care and use of animals in research, which were approved by the Animal Care and Use Committee of Jilin University, Changchun, China.

### Sample collection

The tissues of porcine eye were obtained from three female and three male newborn piglets. They were, rinsed in Dulbecco's phosphate-buffered saline (PBS, Sigma, St. Louis, MO, USA), immediately frozen in liquid nitrogen and stored in −80 °C until use.

The protocol for cell harvesting was previously described in detail (Han *et al*., 2013; Wang *et al*., 2014b). Briefly, matured eggs were electrically and parthenogenetically activated (PA) by two direct current pulses of 1.2 kV/cm for 30 μs using a BTX Electro Cell Manipulator 2001 (BTX, San Diego, CA) and then cultured in cytochalasin B to suppress the extrusion of the second polar body. PA embryos were then transferred into the oviduct of a surrogate sow on the next day. PA fetuses were collected from each uterine horn at day 28.

### Gene expression analysis

Total RNA was isolated from porcine eye cells using the TRNzol reagent (TIANGEN, Beijing, China) according to the instructions of the manufacturer. The RNA was treated with DNase I (Fermentas) and reverse transcribed to cDNA using the BioRT cDNA first strand synthesis kit (Bioer Technology, Hangzhou, China). Quantitative real-time PCR was performed to determine the expression of *H19* and *IGF2*. The primer sequences are listed in Table 1. Quantitative gene expression analysis was carried out according to the manufacturer's instructions using the BIO-RAD iQ5 Multicolor Real-Time PCR Detection System with the BioEasy SYBR Green I Real Time PCR Kit (Bioer Technology, Hangzhou, China). The thermal cycling conditions were 95 °C for 3 min, followed by 40 cycles of denaturation at 95 °C for 10 s, annealing at 55 °C for 15 s, and extension at 72 °C for 30 s. The 2^-ΔΔCT^ formula was used to determine relative gene expression, which was normalized to the quantity of *GAPDH* mRNA. All experiments were repeated three times for each gene. Data are expressed as means ± S.E.M.

**Table 1:** Primers for qRT-PCR analysis.

Genes	Annealing (°C)	Primer sequences (5′ → 3′)	Size (bp)	Reference/accession
*H19*	55	F: CTCAAACGACAAGAGATGGT	122	(Park *et al*., 2011)
		R: AGTGTAGTGGCTCCAGAATG		
*IGF2*	55	F: AAGAGTGCTCTTCCGTAG	156	(Park *et al*., 2011)
		R: TGTCATAGCGGAAGAACTTG		
*GAPDH*	55	F: ATTCCACGGCACAGTCAAGG	120	NM_001206359.1
		R: ACATACTCAGCACCAGCATCG		

### Methylation pattern of H19 DMR3, IGF2 DMR1/2

The procedure for BSP was previously described by Clark *et al*. (1994). Briefly, genomic DNA of porcine eye cells was isolated using the TIANamp Genomic DNA Kit (TIANGEN, Beijing, China) and treated with the CpGenome Turbo Bisulfite Modification Kit (Millipore) according to the manufacturer's instructions. Nested PCR using Taq Plus PCR Master Mix (TIANGEN, Beijing, China) was performed for the amplification of the *H19* DMR3 and *IGF2* DMR1/2. The primer sequences are listed in Table 2. PCR products were purified and subjected to BSP (10 positive clones) and Combined Bisulfite Restriction Analysis (COBRA), which have been described in a previous study (Watanabe *et al*., 2010; Huntriss *et al*., 2013).

**Table 2:** Primers for bisulfite sequencing PCR analysis.

Sequence	Annealing (°C)	Primer sequences (5′ → 3′)	Size (bp)	Reference/
				accession
*H19* DMR1	55 °C	Outer F:AGGAGATTAGGTTTAGGGGAAT	260	(Park *et al*., 2011)
		R: CTACCACTCCCCTCATACCTAA		
		Inner F: AGTGTTTGGGGATTTTTTTTTT		
		R: CACCCCATCCCCTAAATAACCCTC		
*H19* DMR2	55°C	Outer F:TATGTTTAGGGGTGATAAAAGT	216	(Park *et al*., 2011)
		R: CCCCACTTCTACAATTCAAC		
		Inner F: AGGTGTTATTTTGTTTGTTGGT		
		R:ATAAAATAACCTAAAAAAACTCAA		
*H19* DMR3	55 °C	Outer F: GGTTTTAGGGGGATATTTTTT	208	(Park *et al*., 2011)
		R:TTAAAAAAACATTACTTCCATATAC		
		Inner F: GATTTTTAGGTTTGTTATTATTT		
		R: CAAATATTCAATAAAAAAACCC		
*IGF2* DMR1	55 °C	Outer F:GGAAGTTTTGTTTAGTTGGTTTTT	389	(Braunschweig *et al*., 2011)
		R:AAATCTAAAAACAAAAACAAAAAAC		
		Inner F:GTTAGGTTTAGTGTTTAGTATTGGTTT		
		R: TCCAAAACCAAACCTCTCCTAC		
*IGF2* DMR2	55 °C	Outer F:GTTAGGGGGGGGTTTGGTTTTTTAG	268	(Braunschweig *et al*., 2011)
		R: CTCCCCTTAATCCTATAAAACTTCC		
		Inner F: GGTAGTATTTGAAGTTTAAGAG		
		R: CTATAAAACTTCCAAACAAACC		

#### Statistical analysis

The obtained qRT-PCR and BSP data were analyzed by *t*-tests using SPSS 16.0 software (SPSS Inc., Chicago, IL, USA). A value of *p* < 0.05 was considered statistically significant. The methylation status was analyzed using the online BiQ Analyzer software (http://biq-analyzer.bioinf.mpi-inf.mpg.de/tools/MethylationDiagrams/index.php).

## Results

### Imprinted gene expression analysis of *H19* and *IGF2*

**Figure 1:**
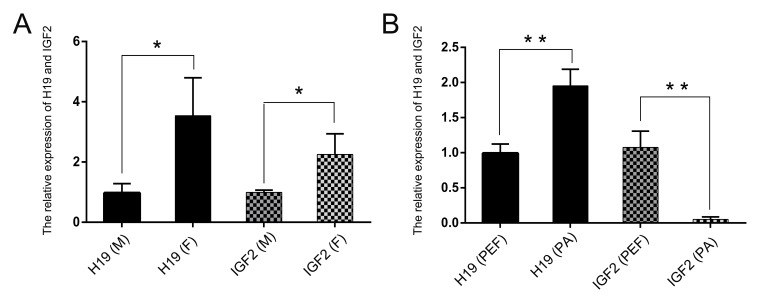
Relative expression levels of *H19*/*IGF2*. qRT-PCR analysis of *H19*/*IGF2* in (A) male and female eye, (B) porcine embryonic fibroblast (PEF) and parthenogenetic (PA) cells. F: female; M: male. Data are reported as means ± SEM (n = 3). * *p* < 0.05, ** *p* < 0.01.

The qPCR results showed that the expression of *IGF2* was up-regulated 2-fold in female eyes compared with male eyes. Furthermore, *H19* expression was also up-regulated 3-fold (Figure 1A).

In order to investigate if the elevated expression was regulated by a specific chromatin structure in *H19*/*IGF2* clusters in female eyes, PA cells were analyzed using qPCR. As expected, the expression of *H19* in PA cells was up-regulated, while *IGF2* expression was significantly reduced compared with porcine embryonic fibroblast (PEF) cells (Figure 1B).

### Methylation analysis of *H19* DMR3 and *IGF2* DMR1/2

**Figure 2:**
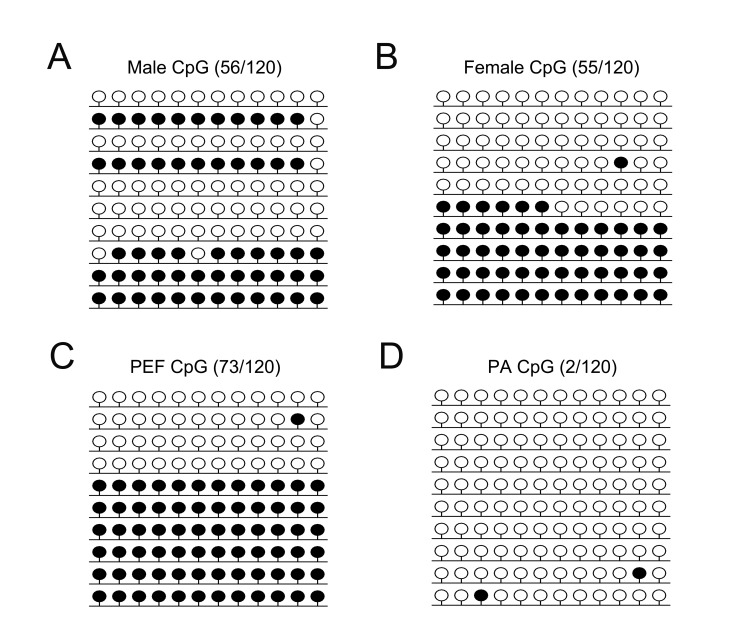
Methylation pattern of *H19* DMR3. CpG methylation profiles of *H19* DMR3 in male eye (A), female eye (B), porcine embryonic fibroblast (PEF) cells (C) and parthenogenetic (PA) cells (D). The black and white circles indicate methylated and unmethylated CpGs, respectively.

The methylation level of *H19* DMR3 was lower in the female eye compared to the male eye (45.2 ± 0.8% and 51.3 ± 4.4%, respectively) (Figure 2A,B). The PCR products were subjected to COBRA analysis, which confirmed our results (Figure S1A). Statistical analyses confirmed the significant difference (Figure 4A). These results suggested that the *H19* DMRs were hemi-methylated in both female and male eyes.

**Figure 3:**
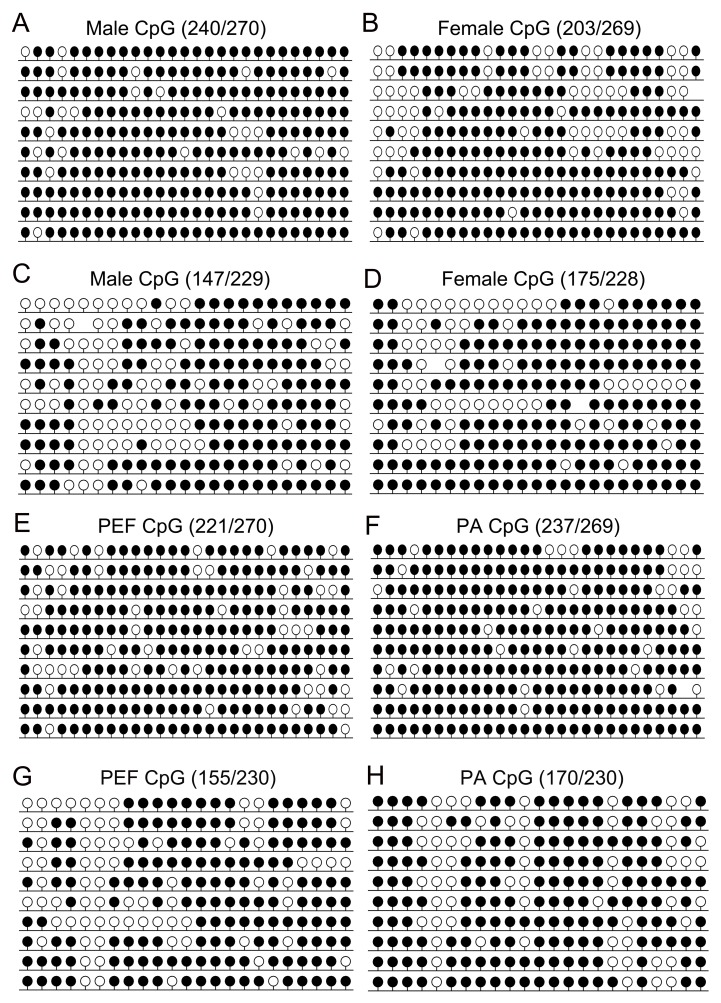
Methylation pattern of *IGF2* DMRs. CpG methylation profiles of *IGF2* DMR1 and DMR2 in (A-D) male eye and female eye, (E, G) porcine embryonic fibroblast (PEF) cells (F,H) parthenogenetic (PA) cells. The black and white circles indicate methylated and unmethylated CpGs, respectively.

**Figure 4:**
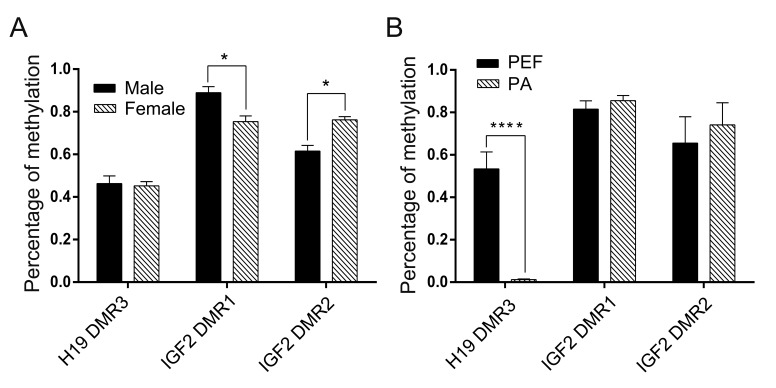
Percentage of methylated CpG sites within *H19* DMD and *IGF2* DMRs between male and female eyes (A), and between porcine embryonic fibroblast (PEF) and parthenogenetic (PA) cells (B). * *p* < 0.05, *** *p* < 0.005.

Compared with *H19* DMRs, *IGF2* DMR1/2 was found to be the hypermethylated in porcine eyes. The results of BSP showed that 75.4 ± 1.1% of *IGF2* DMR1 were hypomethylated in the female eye compared with 89 ± 1.1% in the male eye (Figure 3A,B), while 76.3 ± 0.6% of *IGF2* DMR2 were hypermethylated in the female eye compared with 64.1 ± 1% in the male eye (Figure 3C,D). The PCR products were subjected to COBRA analysis, which confirmed the results (Figure S1B). Statistical analyses revealed that there was a significant difference in *IGF2* DMR1 and DMR2 between female and male eyes (Figure 4A). Taken together, these results suggested that the *IGF2* DMRs methylation status play a crucial role in the regulation of imprinted gene expression in the female eye.

### Methylation status analysis of PA cells

**Figure 5:**
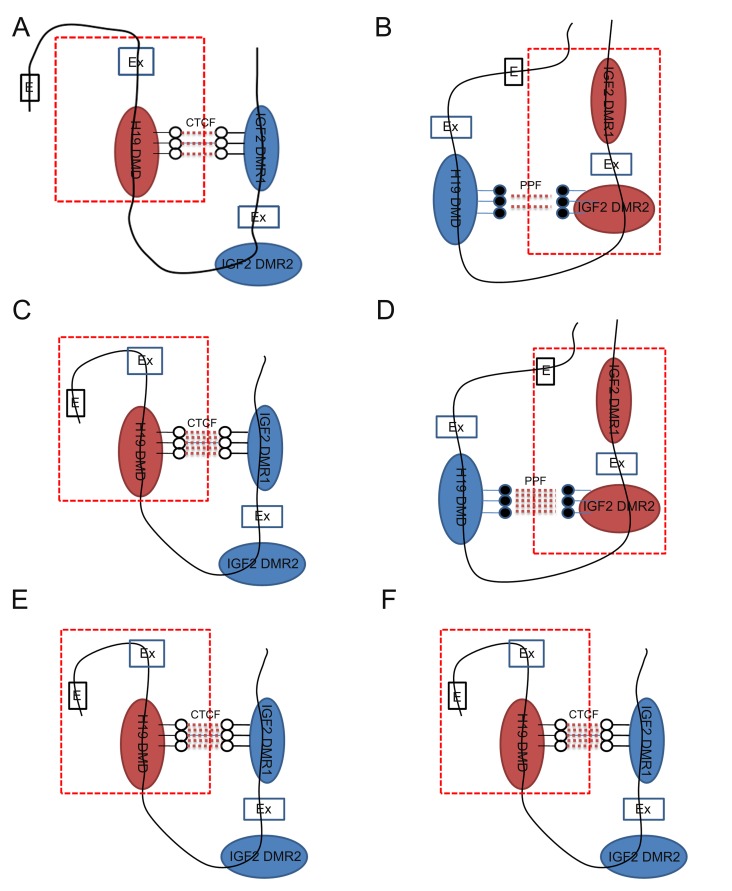
Schematic representations of *H19*/*IGF2*. DNA methylation interactions of *H19* DMD and *IGF2* DMRs on the maternal (A) and paternal allele (B) in the male eye. The specific chromatin structure is described on the maternal (C) and paternal allele (D) in the female eye. As expected, the specific chromatin structures are established as in parthenogenetic (PA) cells (E and F). Ex: exon; E: enhancer; Red dotted line: active domain; CTCF: CCCTC-binding factor - binding sites; PPF: putative protein factors.

The expression pattern and methylation status of *H19*/*IGF2* led us to hypothesize that a specific chromatin structure was located in the porcine female eye (Figure 5). To further investigate whether the expression of *H19* and *IGF2* were associated with their respective DMRs patterns, *H19* DMRs and *IGF2* DMRs were analyzed in PA cells. The BSP data showed that *H19* DMRs in PA cells were significantly demethylated (*p* < 0.05) compared with those in PEF cells (Figure 2C,D), while *IGF2* DMR1/2 were found to be hypermethylated in both PA and PEF cells (Figure 3E-H). The PCR products were subjected to COBRA analysis, which confirmed our BSP results (Figure S1C,D). Statistical analyses revealed that there was no difference in the methylation status of *IGF2* DMR1 and DMR2 between PA and PEF cells (Figure 4B). Taken together, our results demonstrate that the hypomethylated patterns of DMRs regulated the *H19* over-expression in maternal chromatin. These results indicate that the interaction of DNA methylation could change chromatin structure to regulate gene expression in *H19*/*IGF2* clusters.

## Discussion

The chromatin-loop model was first developed to account for *H19*/*IGF2* imprinting expression by *IGF2* DMRs (Murrell *et al*., 2004). Usually, the *IGF2* DMR1 moves to an active domain, while *IGF2* DMR2 moves to an inactive domain on the maternal allele. When *H19* DMRs and *IGF2* DMR1 are both unmethylated, *H19* promoters in close proximity become enhancers. This interaction between the *H19* DMRs and *IGF2* DMR1 results in *H19* expression. In contrast, when the *IGF2* DMR2 and DMR1 both move to an active domain on the paternal allele, *H19* DMRs interacts with *IGF2* DMR2. Because the *H19* DMRs are methylated, *IGF2* promoters come close to enhancers, resulting in *IGF2* expression. In this model, *IGF2* DMRs play an important role in the regulation of *H19* and *IGF2* expression. In the present study, the results of our BSP analysis suggested that there was a specific chromatin structure that might be regulated by DNA methylation in the porcine female eye. Compared with the male eye (Figure 5A,B), hypomethylated *IGF2* DMR1 closely interacts with *H19* DMRs, and thus forces *H19* over-expression on the maternal chromosome (Figure 5C). Compared with the male eye, hypermethylated DMR2 of *IGF2* closely interacts with *H19* DMRs on the paternal chromosome in the female eye. This forces *IGF2* over-expression (Figure 5D).

Due to the fact that *H19* DMRs were found to be hemi-methylated in both male and female eyes, we explored whether the hypomethylation status of DMRs closely interacted with each other on the maternal chromosome. In this study, we used PA cells to determine if the elevated expression of *H19*/*IGF2* in the porcine female eye was regulated by the DNA methylation interaction. Indeed, evolutionarily conserved sexually dimorphic expression of *H19/IGF2* suggests a functional sex difference. PA cells contain exclusively maternal chromatin, thus, *H19*/*IGF2* expression from paternal chromatin was not a confounding factor in the analyses (Zhu *et al*., 2003).

In our previous study, BSP results suggested that there was no difference in *IGF2* DMR1 and DMR2 between PA and normal fetuses (Wang *et al*., 2014a). These data are in accordance with the present results from PA cells. In addition, the *H19* DMRs were hypomethylated in PA cells, confirming previous reports of aberrant methylation profiles in oocytes, PA embryos and fetuses (Park *et al*., 2009; Wang *et al*., 2014a). Our results indicate that *H19* DMRs closely interact with *IGF2* DMR1 on the maternal chromosome in PA samples (Figure 5E,F). Due to the hypomethylated DMRs, *H19* expression was higher in PEF cells, while *IGF2* was not expressed. The results from the PA cells indirectly show that *IGF2* was over-expressed by the hypermethylation status of *IGF2* DMR2 on the paternal chromosome in the female eye.

In summary, the results of the present study demonstrate the elevated expression of *H19/IGF2* in female porcine eye, which was associated with the methylation patterns of *IGF2* DMRs. Our detailed analysis of PA and PEF cells suggest that a specific chromatin structure was formed due to the methylation of DMRs, which regulated the expression of *H19*/*IGF2*.

## Supplementary Material

The following online material is available for this article:

Figure S1 - Methylation pattern of *H19* DMD and *IGF2* DMRs.
