# Supplementary material for: DNA methylation modulates H19 and IGF2 expression in porcine female eye
Source: Genet Mol Biol. 2017 Mar 6;40(1):153–9. doi: 10.1590/1678-4685-GMB-2016-0194 (PMC5409778; doi:10.1590/1678-4685-GMB-2016-0194)
Supplement: Supplementary file 1 [file 1415-4757-gmb-1678-4685-GMB-2016-0194-Suppl01.pdf]

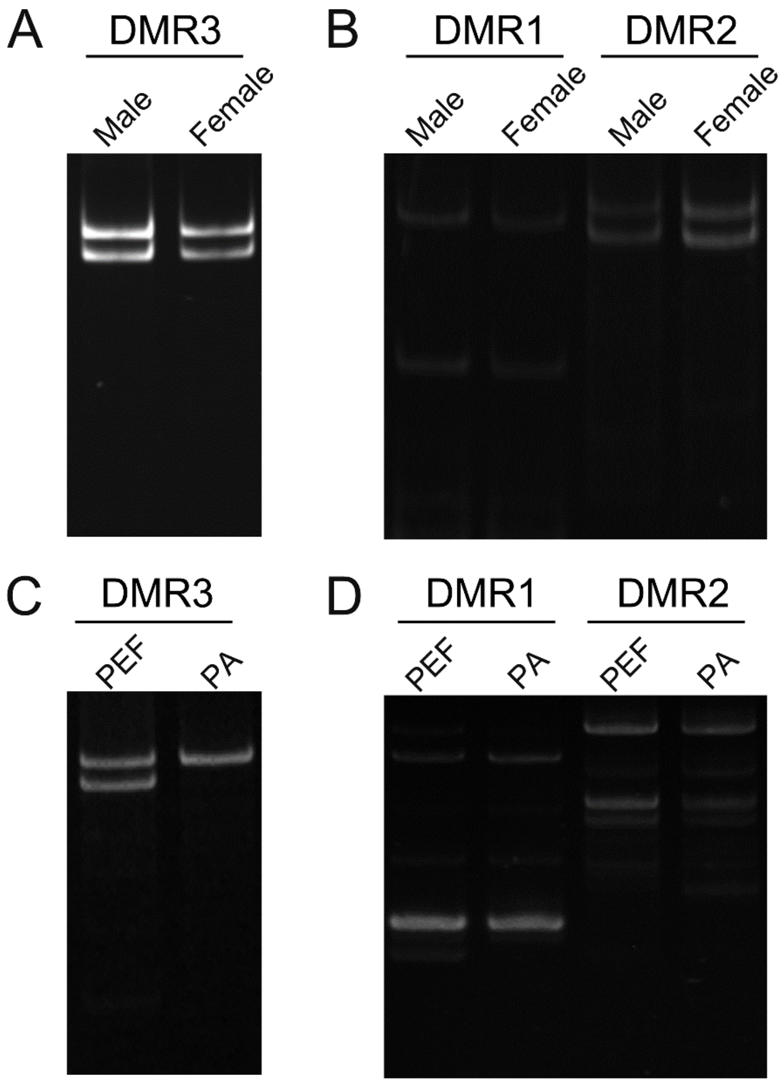

Figure S1 - Methylation pattern of *H19* DMD and *IGF2* DMRs. For COBRA analysis, the PCR products of *H19* DMD and *IGF2* DMRs were digested with the restriction enzyme Taq I in male and female eye (A and B), and porcine embryonic fibroblast (PEF) and parthenogenetic (PA) cells (C and D), respectively.
